# Where the Little Ones Play the Main Role—Picophytoplankton Predominance in the Soda and Hypersaline Lakes of the Carpathian Basin

**DOI:** 10.3390/microorganisms10040818

**Published:** 2022-04-14

**Authors:** Boglárka Somogyi, Tamás Felföldi, Emil Boros, Attila Szabó, Lajos Vörös

**Affiliations:** 1 Balaton Limnological Research Institute, Klebelsberg Kuno Str. 3, P.O. Box 35, H-8237 Tihany, Hungary; voros.lajos@blki.hu; 2 Institute of Aquatic Ecology, Centre for Ecological Research, Karolina Str. 29, H-1113 Budapest, Hungary; tamas.felfoldi@gmail.com (T.F.); boros.emil@ecolres.hu (E.B.); attila.szabo.ttk@gmail.com (A.S.); 3 Department of Aquatic Sciences and Assessment, Swedish University of Agricultural Sciences, Lennart Hjelms Vag 9, 750 07 Uppsala, Sweden

**Keywords:** autotrophic picophytoplankton, abundance, seasonal dynamics, diversity, extreme environments

## Abstract

The extreme environmental conditions of the diverse saline inland waters (soda lakes and pans, hypersaline lakes and ponds) of the Carpathian Basin are an advantage for picophytoplankton. The abundance of picophytoplankton in these waters can be up to several orders of magnitude higher than that in freshwater shallow lakes, but differences are also found within different saline water types: higher picophytoplankton abundances were observed in hypersaline lakes compared to humic soda lakes, and their highest numbers were detected in turbid soda lakes. Moreover, their contribution to phytoplankton biomass is higher than that in shallow freshwater lakes with similar trophic states. Based on long-term data, their ratio within the phytoplankton increased with turbidity in the case of turbid soda lakes, while, in hypersaline lakes, their proportion increased with salinity. Picocyanobacteria were only detected with high abundance (>10^6^–10^7^ cells/mL) in turbid soda lakes, while picoeukaryotes occurred in high numbers in both turbid and hypersaline lakes. Despite the extreme conditions of the lakes, the diversity of picophytoplankton is remarkable, with the dominance of non-marine *Synechococcus/Cyanobium*, *Choricystis*, *Chloroparva* and uncultured trebouxiophycean green algae in the soda lakes, and marine *Synechococcus* and *Picochlorum* in the hypersaline lakes.

## 1. Discovering the Importance of Picophytoplankton in the Soda and Saline Lakes of the Carpathian Basin

While the study of the smallest algae (photo**a**utotrophic **p**ico**p**lankton (APP), also referring to picophytoplankton covering the size range between 0.2 and 2 or 3 µm of picocyanobacteria and picoeukaryotes) began before the turn of the millennium in most continental waters, it happened later in the case of soda and hypersaline lakes. In the soda lakes of the Carpathian Basin, picophytoplankton were first studied in the 2000s, and in the hypersaline lakes a decade later (Table S1). Despite the late start, due to the intensity of research, ‘lagging’ has quickly disappeared and a number of studies on the picophytoplankton community of these extreme habitats have been published to date. What makes these waters so special in terms of picophytoplankton is their relative monopoly. In many cases, only picophytoplankton occurs within the phytoplankton, which is certainly related to the extreme physical and chemical conditions of the lakes. We will review this topic in the following, summarizing all the studies that have been published so far, and complete them with a meta-analysis of the available data.

The discovery of the importance of picophytoplankton in the extreme aquatic habitats of the Carpathian Basin is linked to the turbid soda lakes, where their high abundance and biomass have been described [1]. Studies of the planktonic picophytoplankton communities of humic soda lakes only started subsequently [2]. For both lake types, the studies were initially carried out in the period from spring to autumn, and only later were expanded with winter surveys. Based on the pigment types, two distinct groups were identified in soda lakes: phycocyanin pigment-dominated single-celled picocyanobacteria with a 1–2 µm diameter and single-celled picoeukaryotic algae with a 2–3 µm cell size [1,3,4,5,6,7,8,9,10]. Neither phycoerythrin nor colonial forms have been observed to date. The only exception occurred in a large soda lake, Lake Neusiedl, where colonial picocyanobacteria were detected with high abundance in the open water [11,12]. Molecular taxonomic studies also began in the early 2000s, studying both environmental samples (by denaturing gradient gel electrophoresis (DGGE), cloning and next-generation DNA sequencing [2,8,11,13]) and isolated picocyanobacterial [14] and picoeukaryotic strains [9,15,16].

In hypersaline lakes, picophytoplankton studies were first performed in 2010, where microscopic and molecular taxonomic work started simultaneously [17]. These lakes were not regularly surveyed as soda lakes, but occasional samplings were carried out between 2010 and 2015 (examining about 60 samples of a total of 9 hypersaline lakes; Table S2). Therefore, we represented these hypersaline lakes as a single group in the figures. Similar to the soda lakes, in hypersaline lakes, phycocyanin-rich picocyanobacteria and picoeukaryotes were present in high numbers, constituting a notable part of the total phytoplankton biomass [6,7]. The diversity of the APP community was studied by DGGE [17,18] and the cloning [7] of environmental samples. Picoeukaryotic strains were also isolated and identified by DNA sequencing (see below).

## 2. Extreme Conditions—Physical and Chemical Environment in Soda and Saline Lakes of the Carpathian Basin

The Carpathian Basin is rich in athalassohaline water bodies; considering their water chemistry features, they are divided into two distinct types. Soda lakes with sodium carbonate dominance represent one of the most unique water bodies in Europe occurring in Austria, Hungary and Serbia [19], while numerous (41) saline lakes with sodium chloride dominance have been charted in the Transylvanian Basin (Romania) formed on Miocene halite deposits [20]. Due to their unique conditions and biota, as well as the significant decline in their number and territory, soda lakes (n = 84) are listed in the habitat directive (92/43/EC) as “Pannonian steppes and salt marshes” with high protection priority in the Natura 2000 network of the European Union. Several of these habitats are listed as Ramsar sites or Important Bird Areas, and most soda lakes in Austria are part of a UNESCO World Heritage site [21]. There are several different limnological types of standing waters in this review (Figure S1). The largest water body is Lake Neusiedl, which is a semistatic shallow alkaline soda lake. A number of intermittent alkaline soda pans were also included in this study, which are situated in the central region of Hungary (Danube–Tisza interfluve area) and in Serbia (Vojvodina). The third type of standing waters are permanent deep saline ponds in Romania (Transylvania). Hereinafter, all these different limnological types of inland standing waters are referred to as ‘lakes’ to simplify terms through the study.

Most of the soda lakes in this region are very shallow (depth~15 cm) and quite small (surface area up to 500 ha), with the exception of two large lakes (Lake Neusiedl and Lake Velence), which are more than 1 m deep and have surface areas of 309 and 24 km^2^, respectively [22]. Concerning their chemical composition, the amount of calcium (Ca^2+^) and magnesium (Mg^2+^) ions are low, while sodium (Na^+^) and carbonates (HCO_3_^−^ + CO_3_^2−^) are the dominant (>25 equivalent percentage) ions in the water. Soda lakes represent the most stable permanent high-pH environments (pH > 9) on Earth, which clearly distinguishes them from other inland saline waters [22]. The salinity varies between the sub- and the mesosaline range (1–27 g/L), with a median value of 3 g/L in the hyposaline range [19].

Due to their shallowness, the presence of large amounts of clay and other inorganic particles causes high turbidity and, as a result, the colour of the water is often light grey (Table S2, Figure S2). Lakes in the absence of large amounts of suspended solids but having significant amounts of dissolved humic substances appear brown-coloured [23]. In the present study, a distinction was made between turbid and humic soda lakes according to Boros et al. [21].

Based on the annual average total phosphorous concentration (Table S2), the overwhelming majority of soda lakes can be classified as hypertrophic systems according to the OECD (1982) classification. The observed nitrogen and phosphorus ratio is often extremely low (N/P < 1), because high water temperatures, continuous mixing, resuspension and alkalinity promote significant NH_3_ emanation losses from these soda lakes [24]. The characteristic environmental features of soda lakes, such as the combination of shallowness, intermittent character (periodic desiccation), high turbidity, high concentration of dissolved humic substances, hypertrophic nutrient concentration, high daily water temperature fluctuation, and high alkalinity altogether create a multiply extreme environment [25].

The hypersaline lakes of the Transylvanian Basin (Romania, eastern part of the Carpathian Basin) are artificial water bodies with Na^+^ and Cl^−^ ion dominance contributing to >90% of the total salt content with surface areas between 0.1 and 4 ha formed by the collapse and inundation of abandoned salt mines [20,26]. Most of these lakes are affected by a notable anthropogenic impact, as the majority of them are popular bathing resorts. In contrast to the small surface area, their depth varies from 1 to 100 m. The deep lakes of this region are meromictic with characteristic permanent density stratification. The mixing occurs in the moderately saline upper stratum (mixolimnion), and the bottom layer (monimolimnion) is stagnant and hypersaline [27]. Some of them show a rare phenomenon called heliothermy, a unique thermal profile with a double stratification between the spring and autumn: increasing temperature to the depth of 1.5–3.5 m (maximum 35–45 °C), and decreasing temperature from the thermal maximum to the bottom. In these water bodies, dramatic changes in the physical and chemical properties occur within a short distance, resulting in the formation of distinct microhabitats [28].

## 3. Extremely High Picophytoplankton Abundance in the Soda and Saline Lakes of the Carpathian Basin

**Figure 1:**
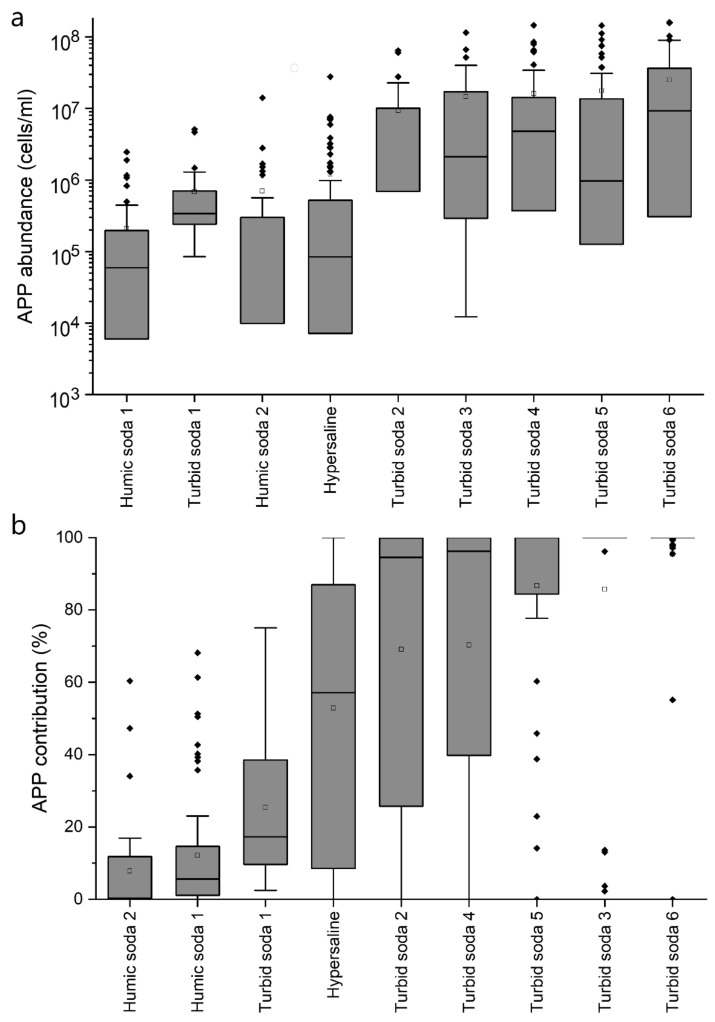
Abundance (**a**) and contribution (**b**) of picophytoplankton (APP) to the total phytoplankton biomass in the studied soda and hypersaline lakes (re-analysis of data from references detailed in Table S1, expanded with unpublished results).

The abundance of picophytoplankton is affected by a number of factors from bottom–up (e.g., nutrient content and amount, light conditions and temperature) to top–down (grazing and viral lysis) effects. In the case of the soda and hypersaline lakes studied, the APP abundance varied within a wide range, practically in four orders of magnitude (between 10^4^ and 10^8^ cells/mL). The lowest APP abundance values were found in humic soda lakes, with mean values ranging from 2 to 7 × 10^5^ cells/mL (Figure 1, Table 1). Higher values were found in hypersaline lakes, where the mean APP abundance ranged from 0.1 to 24 × 10^5^ cells/mL. The APP abundances were highest in turbid soda lakes, averaging between 7 and 26 × 10^6^ cells/mL (Table 1). The maximum values were remarkably high (1–1.6 × 10^8^ cells/mL), probably the highest APP abundance values reported in the world (the extremely high values reported so far are about one order of magnitude lower, see [4,5,8,29,30]. In terms of seasonality, these lakes are characterized by high APP biomass, not just during the summer, but also during the winter. Even winter algal blooms have been observed in turbid soda lakes formed by picophytoplankton only [5,8]. Regarding the composition of the picophytoplankton, two cells type can be distinguished by microscopy: phycocyanin-rich picocyanobacteria, which are usually described from eutrophic waters and picoeukaryotic algae, which are characteristic of winter phytoplankton communities in shallow lakes. Differences were observed in the occurrence of the two groups in different saline water bodies (Figure S3). The average abundance of picocyanobacteria (CyAPP) in hypersaline lakes (1.5 × 10^5^ cells/mL) and humic waters (2–5 × 10^5^ cells/mL) was relatively low, and the highest values were observed in turbid soda lakes (0.1–1.2 × 10^7^ cells/mL). Picoeukaryotes (EuAPP) were not detected at all in one lake (Lake Neusiedl), and only occasionally in the humic soda lakes, with low average abundance values (1–20 × 10^4^ cells/mL). The abundance of picoeukaryotes was somewhat higher in hypersaline lakes (8 × 10^5^ cells/mL on average), while the highest average EuAPP abundances were observed in turbid soda lakes (0.1–2 × 10^7^ cells/mL), similar to CyAPP.

It is a generally accepted trend that the abundance of picophytoplankton increases with the trophic state [31,32,33,34]. Bell & Kalff [33] reported regression models describing this relationship in oceans and deep lakes, and we previously reported similar equations for shallow lakes [10]. In the case of shallow lakes, two groups were distinguished based on their TSS content: shallow lakes with a TSS concentration lower than 50 mg/L and a higher TSS concentration, as the TSS content was shown to significantly affect the abundance of picophytoplankton [10]. In fact, the latter group mostly included turbid saline lakes, so the equation for lakes with a TSS content lower than 50 mg/L can also be considered as a valid relationship for freshwater shallow lakes. Regarding the soda and hypersaline lakes of the Carpathian Basin, a significant positive relationship was found between the chlorophyll *a* concentration and APP abundance in all three lake types (Table 2, Figure S4). The slope and intercept of the regression models were significantly different from those described earlier for deep and shallow freshwater lakes (Table 2). The slope of the regression equation for turbid soda lakes was significantly greater than that for hypersaline lakes (Table 2, Figure S4). The reason for this difference is probably the TSS content, as found earlier when comparing shallow lakes [10].

**Figure 2:**
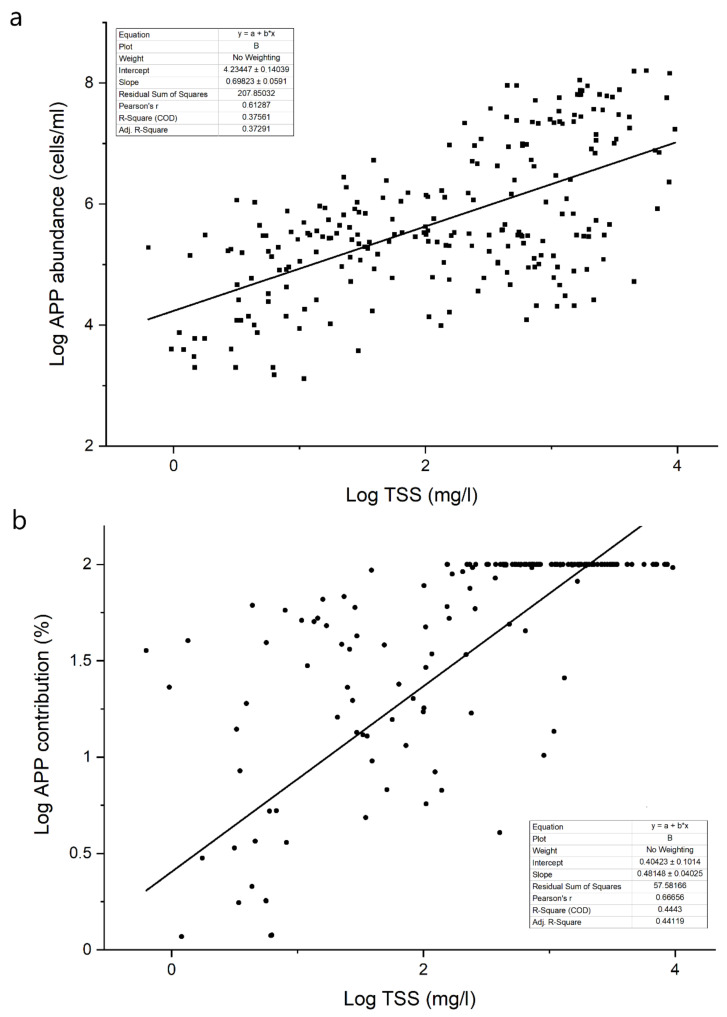
Relationship between the APP abundance (**a**) or contribution (**b**) and TSS concentration (**a**) in the studied soda and hypersaline lakes (re-analysis of data from the references detailed in Table S1, expanded with unpublished results).

Regarding soda lakes, a significant positive relationship was found between the TSS concentration and APP abundance (Figure 2). The TSS concentration fundamentally affects grazing, the most important top–down factor of APP dynamics [10]. Generally, the grazing pressure on APP is higher than that on larger-sized phytoplankton, because heterotrophic flagellates, ciliates, rotifers and microcrustaceans are all able to consume APP [35]. In addition, the major consumers of APP (protozoa) have a shorter generation time than consumers of nano- and microplankton (metazoa). As a result, grazing control on APP is much tighter than that on larger organisms [36,37]. Thus, the development of APP mass production assumes a lack of grazing control, which may be due to several reasons, but one of the most obvious in the present case is the effect of TSS.

Decreasing grazing pressure as a result of increasing turbidity is usually explained by the relative decrease of available food (i.e., phytoplankton) as compared to non-ingestible inorganic particles. High abundances of such particles over the size range of potential food particles can lead to reduced population growth rates or even the death of zooplankton. When the suspended sediment concentration exceeds the threshold below which zooplankton species can effectively filter material, the organisms could starve, even with high food abundance [38]. Several studies have demonstrated the decline of zooplankton feeding with increasing TSS. In the case of *Daphnia galeata,* a threshold of 25 mg/L was found, above which the clearance rate decreased [39]. A similar phenomenon was described during feeding experiments with five cladoceran species [40,41] as well as in the case of a fairy shrimp [42]. A high TSS content also inhibited the filtration of a rotifer *Brachionus calyciflorus* and growth of the ciliate *Strobilidium gyrans* [43,44].

## 4. Success of Picophytoplankton as the Environment Becomes Extreme

It is widely accepted that the relative importance of picophytoplankton (their percentage in total biomass and primary production) decreases with increasing trophic status [33,34]. Similar to the APP abundance, empirical models have been described for oceans and deep lakes to quantify the negative relationship between the APP contribution and chlorophyll *a* [33]. In the case of the soda and hypersaline lakes studied, the APP contribution varied between 0 and 100%. The lowest average contribution (8–11%) was found in humic-rich waters, higher values (0.3–90%) in hypersaline lakes, and the highest average APP contribution (25–95%) was found in turbid soda lakes (Figure 1, Table 1). However, there was no significant correlation between the chlorophyll *a* concentration and APP contribution in either case (Figure S5). Regardless of trophic state, APP often reached 100% dominance in hypersaline lakes, and the same was observed in turbid soda lakes (Figure S5). Comparing the present data with the previous equations described for deep and shallow lakes, the APP contribution was significantly higher in hypersaline lakes and turbid soda lakes than in shallow and deep freshwater lakes with similar trophic states (Figure S5). In the case of humic soda lakes, a clear trend was not observed between chlorophyll *a* and APP contribution (Figure S5).

In soda lakes, the contribution of picophytoplankton to total phytoplankton biomass increased with the TSS concentration (Figure 2). The higher APP abundance and contribution values found in soda lakes indicated that picophytoplankton have a selective advantage over larger algae in aquatic habitats with high TSS contents. As described in the previous chapter, the lack of grazing control may be a good explanation for the extremely high planktonic picophytoplankton abundances; however, other factors could also contribute to the success of picophytoplankton. A high TSS concentration results in strong light limitation, and picophytoplankton have a selective advantage over larger phytoplankton under light-limited conditions [9,45,46,47,48]. Shallow, turbid, saline lakes have been previously described as highly light-limited aquatic habitats: the average Z_mix_/Z_eu_ ratio in these waters was 2.8, which still allowed net photosynthesis (the critical value is above 5–5.7), but this ratio was significantly higher than that measured in shallow lakes with TSS below 50 mg/L [10].

The selective advantage of picophytoplankton is due to the reduced chromophore self-shading associated with a smaller cell size, which makes light harvesting at low photon flux densities more efficient [49]. A low-light-adapted APP strain can have a light-saturation parameter as low as 3 μmol/m^2^/s [45]. Such low values were not observed for algal strains isolated from turbid soda lakes: the light-saturation parameter of the studied strains varied between 30 and 150 μmol/m^2^/s, depending on the temperature [5]. However, during a study on the photosynthesis of natural phytoplankton communities, we found a large difference between turbid lakes dominated by picophytoplankton and shallow lakes dominated by nanoplankton. In the former, the light-saturation parameter of the phytoplankton varied between 50 and 90 μmol/m^2^/s during spring–summer measurements, and remarkable light inhibition was already observed at ~150 μmol/m^2^/s [50]. On the other hand, in the nanoplankton-dominated shallow lakes, the light-saturation parameter of the phytoplankton varied between 80 and 120 μmol/m^2^/s, and no light inhibition was observed up to 500–600 μmol/m^2^/s [50].

Since extreme TSS values were not detected, extremely high APP abundance and contribution were not a result of high TSS in hypersaline lakes. However, these lakes have a feature that can also lead to grazing inhibition: their exceptionally high salinity. Previous studies suggest that grazing pressure may decrease as salinity increases: the abundance of HNFs and ciliates decreased with increasing salinity in solar saltern systems in Spain, disappearing around 25% salt content [51]. We have shown that heterotrophic nanoflagellates (HNFs), the main consumers of APP, are absent from the hypersaline lakes studied [6]. In the hypersaline lakes of the Eastern Tibetan Plateau, Wu et al. [52] also could not find HNFs, despite the high-sensitivity molecular biological methods used. However, *Artemia salina*, a characteristic crustacean in hypersaline lakes, is also able to consume APP [53], but we do not have detailed information on its grazing efficiency. Grazing experiments along a salinity range would be necessary to quantify top–down processes in hypersaline lakes, which might serve as an explanation for the high APP biomass and contribution.

The contribution of APP to phytoplankton biomass is likely proportional to their contribution to primary production. In hypersaline and turbid soda lakes, where APP are predominant, these tiny organisms could be one of the main food sources for zooplankton. Both hypersaline and intermittent soda lakes are special aquatic ecosystems that are not inhabited by massive resident vertebrates (fish, amphibians and reptiles); therefore, zooplankton are at the top of the aquatic food web [21,28]. In these relatively simple food webs, nutrient cycling and the energy flow are faster than those in an average aquatic system. Besides this, heterotrophic and photoheterotrophic bacteria are also present with high abundances in soda lakes [4,54] due to the high primary production of algae and macrophytes and the high organic carbon load of aquatic birds [24,55,56,57]. Thus, in addition to APP, bacterioplankton may also be an important food for zooplankton. It has not been studied whether heterotrophic bacterioplankton or photoautotrophic picoplankton are the main food source for zooplankton in these alkaline, saline ecosystems, but the metabolic balance of lake processes suggests that there may be a difference between humic and turbid saline lakes in this regard. Humic soda lakes are dominated by chemoorganotrophic processes, i.e., the respiration of bacterioplankton and zooplankton significantly exceeds the production of phytoplankton on an annual basis [4,57]. However, the phytoplankton (mainly APP) productivity in turbid soda lakes is significantly higher than that in humic soda lakes [4,57], suggesting that picoalgae are more important food sources for zooplankton in the former than in the latter.

## 5. Factors Determining the Composition of Picophytoplankton in Soda and Saline Lakes

### 5.1. Temperature

EuAPP and CyAPP cells are considerably different in terms of structure and physiology. Generally, low temperatures and light-limited conditions are more favourable for EuAPP than CyAPP, as EuAPP cells have lower light demands and acclimate better (e.g., with their fatty acid content) to low temperatures [8,58], although CyAPP also include low-light-adapted microorganisms [49,59]. In the large lakes of the Carpathian Basin (Lake Balaton and Lake Neusiedl), EuAPP-dominated winter communities have much lower light demands than the CyAPP-dominated summer communities [9].

**Figure 3:**
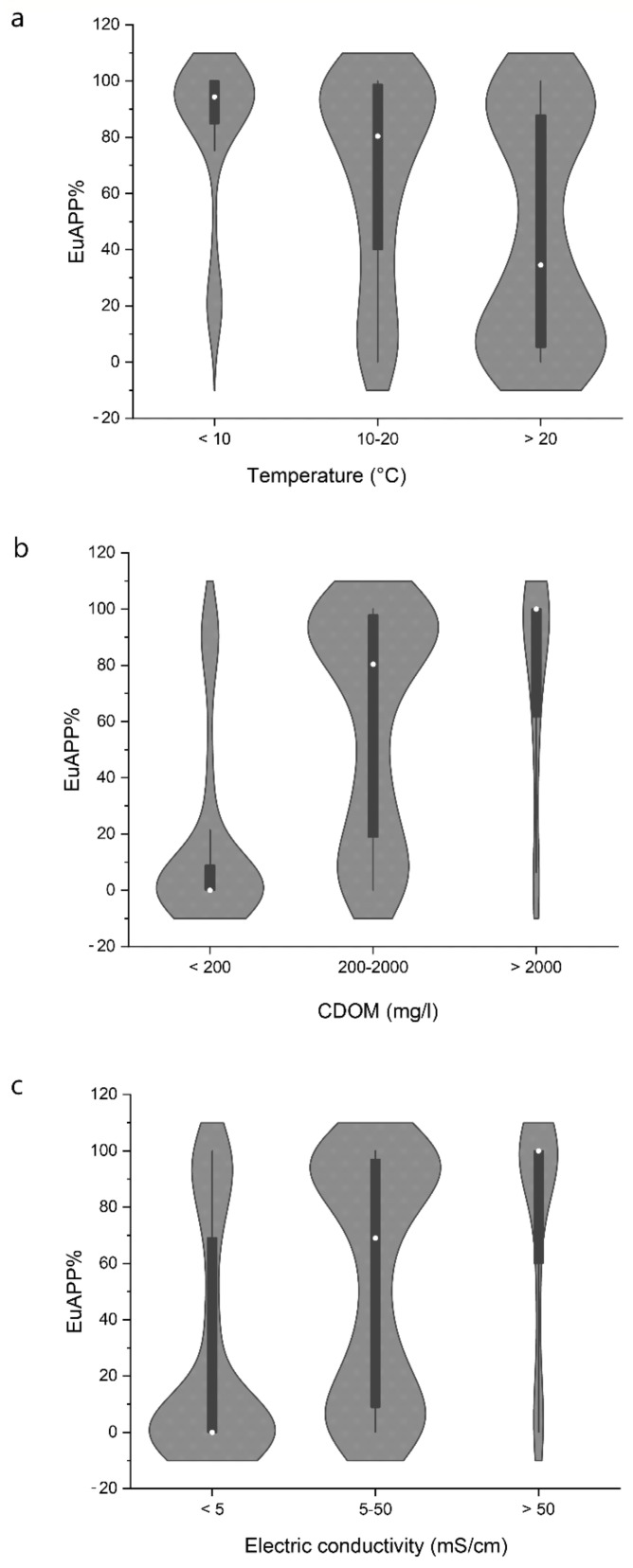
Contribution of picoeukaryotic algae (EuAPP) to the total picophytoplankton along with the temperature (**a**), coloured dissolved organic matter (CDOM) concentration (**b**) and electric conductivity (**c**) (re-analysis of data from the references detailed in Table S1, expanded with unpublished results). The average values are represented by white dots.

In the studied soda and hypersaline lakes, the CyAPP abundance and biomass were the lowest in winter, while higher values were observed in most lakes in the spring, and the maximum values were observed in the summer. The abundance and biomass of EuAPP showed the opposite trend, and the maximum abundance was observed in the winter in most lakes. In turbid soda lakes, a winter picoeukaryotic algal bloom is a recurring phenomenon [5,8]. The contribution of picocyanobacteria and picoeukaryotes to the biomass of the total picophytoplankton changed significantly with the water temperature (Figure 3). Picocyanobacteria were the dominant picoplankters under high temperatures (>20 °C) in the summer, while picoeukaryotes dominated under the low-temperature (<10 °C) conditions in the winter. These seasonal dynamics are in agreement with previous observations described for other temperate lakes, including the shallow lakes of the Carpathian Basin [60,61,62,63,64].

To understand the regulatory role of temperature, the photosynthetic activities of picocyanobacterial (a non-marine *Synechococcus*) and picoeukaryotic algal (*Chloroparva pannonica*) strains isolated from a soda lake were compared at different temperatures and light intensities [5]. The obtained P–I curves help to understand the characteristic seasonal dynamics of these picophytoplankton. Approximately 15 °C appeared to be a turning-point temperature, below which the maximum photosynthetic rate (P_max_) and light utilization parameter (α) of the picoeukaryotic strain exceeded those of the picocyanobacterial strain (Figure S6). The lowest temperature (7 °C) in our experiments, which was close to the midday temperature of the lakes in winter 2006–2007, was not optimal for the picoeukaryotic strain, but its production exceeded that of the picocyanobacterial strain. These results indicated that low winter temperatures provide a competitive advantage to picoeukaryotes. Besides temperature, light is also a significant controlling factor: at low temperatures, picoeukaryotes can utilize low light intensities more efficiently than picocyanobacteria [5]. The winter predominance of picoeukaryotes and the summer predominance of picocyanobacteria seem to be caused by the different light and temperature optima of these groups.

However, there were exceptions to the above described seasonal dynamics between the studied lakes: e.g., in our deepest and largest soda lake (L. Neusiedl), picoeukaryotic algae were not observed in the open water at all, and there were lakes where picoeukaryotes were present in large numbers, even in the summer (hypersaline lakes and humic soda lake 2; Figure S3). In these lakes, the effects of other factors (e.g., salinity and CDOM) were probably more important than the regulatory effect of temperature. This is probably the reason why the relationship between temperature and the abundance/contribution of different types of picophytoplankton was not significant for the whole data set (data not shown).

### 5.2. Brown-Coloured Dissolved Organic Substances (CDOM)

One of the factors that played a significant role in the lakes studied was CDOM. At low CDOM concentrations (<200 mg/L), the dominance of CyAPP was observed, while, above this threshold, the dominance shifted towards EuAPP, and, above 2000 mg/L, we observed almost exclusively EuAPP being dominant within the picophytoplankton (Figure 3). To the best of our knowledge, the contribution of picoeukaryotic algae to the picophytoplankton assemblage has not yet been associated with the CDOM content, although several studies reported the dominance of picoeukaryotic algae in northern humic-rich lakes. Picocyanobacteria were mostly not detected or only observed during a very short time period in many polyhumic Danish, Polish and Finnish lakes [65,66,67].

There are two possible explanations for the observed relationship between CDOM and the algal composition: one is due to the changes in the underwater light climate and the other is due to its inhibitory effect on photosynthesis. The underwater light conditions shift toward red with high CDOM content, because brown-coloured humic substances absorb shorter wavelengths more strongly than longer ones [68]. In turbid and humic soda lakes, the underwater light spectrum does not contain any blue wavelengths. In turbid soda lakes, the dominance of the orange region can be observed, while, in humic soda lakes, red and far-red (>680 nm) light dominates (Figure S7).

Although the light utilization of picophytoplankton at different regions of the light spectrum has been addressed in several studies, none of them have covered the red or the far-red regions of the spectrum. It is widely known that the underwater light quality is the primary cause of the prevalence of phycocyanin rich-CyAPP, which effectively absorb orange–red light (~625 nm) in lakes with orange/red light dominance [69,70,71,72,73,74,75,76,77]. The study of chromatic adaptation in the case of EuAPP has been limited to the blue–orange region, despite the fact that they are dominant in waters that can be characterized by high CDOM content and probably with red light dominance [49,78]. The in vivo absorbance spectra of the two groups showed that CyAPP are efficient in utilizing the orange–red region and EuAPP are able to utilize red-far red light effectively (Figure S7). Therefore, while the former group has a selective advantage in turbid soda lakes, the other group prefer the underwater light spectrum characteristics in humic soda lakes (Figure S7).

Another possible explanation is the inhibitory effect of CDOM on photosynthesis. Humic substances may directly quench electrons or bind to the bioquinones in PSII, thereby blocking electron transfer [79]. Based on previous studies, cyanobacteria appear to be more sensitive to the presence of CDOM than eukaryotic algae, but even closely related species within a taxonomic group may behave differently [79,80]. To the best of our knowledge, picophytoplankton have not been studied in this regard so far, but CDOM may also play a role in shaping the composition of picophytoplankton, as described for nano- and microplankton.

### 5.3. Salinity

There were remarkable differences in picophytoplankton composition along the salinity gradient in the studied soda hypersaline lakes (Figure 3). CyAPP were predominant in less saline waters (mainly below 5 mS/cm conductivity corresponding to ~3 g/L NaCl), but, with increasing salinity, their contribution started to decrease and EuAPP dominance was found above 50 mS/cm (~32 g/L NaCl). Similar to our results, the composition of APP shifted with salinity in a coastal lagoon system: CyAPP was mainly abundant below 3% salinity, while, at salinities ranging from 4.5% to 14.0%, EuAPP was dominant [81]. In a solar saltern system (Tunesia), APP were dominated by EuAPP, with maximum abundances between 7.9% and 19% salinity [82].

The different salinity tolerances of CyAPP and EuAPP were well demonstrated by growth experiments using isolates from the saline, alkaline Lake Mono [53]. A picoeukaryote *Picocystis* strain was able to grow from 0 to 26% salinity, with an optimum at 4% [53], while a picocyanobacterium strain had lower salinity tolerance (growing from 0% to 10% salinity with an optimum at 3%) [83]. Another *Picocystis* isolate from an Inner Mongolian soda lake also exhibited a broad salinity tolerance growing over a salinity range of 2.9–17.5% [84]. The higher salinity tolerance of EuAPP could explain their dominance in hypersaline waters, as was observed in lagoons, hypersaline lakes and solar saltern systems [6,81,82].

The examination of the salt tolerance of picoeukaryotic isolates originating from the freshwater, soda and hypersaline lakes of the Carpathian Basin showed that freshwater and soda isolates were able to grow only in a very narrow salt range (0–30 g/L NaCl containing Johnson’s medium [85]; 0 to 3% salinity). In contrast, *Picochlorum oklahomense* strain ACT1233 isolated from a hypersaline lake was able to grow from 15 to 120 g/L (1.5 to 12% salinity; Figure S8). Henley et al. [86] found similar results when studying the salinity tolerance of a *P. oklahomense* strain originated from the Salt Plains National Wildlife Refuge (Oklahoma, OK, USA). As a result, *Picochlorum* was able to grow from 0 to 10% salinity; however, it exhibited a decreasing growth rate with increasing salinity [86]. According to the literature, there are two picoeukaryotic algae with exceptionally broad salt tolerance: *Picocystis* and *Picochlorum*, which can tolerate much higher salt concentrations than other picoeukaryotic or picocyanobacterial taxa.

## 6. Taxonomic Composition of APP Communities in the Soda and Saline Lakes of the Carpathian Basin

**Figure 4:**
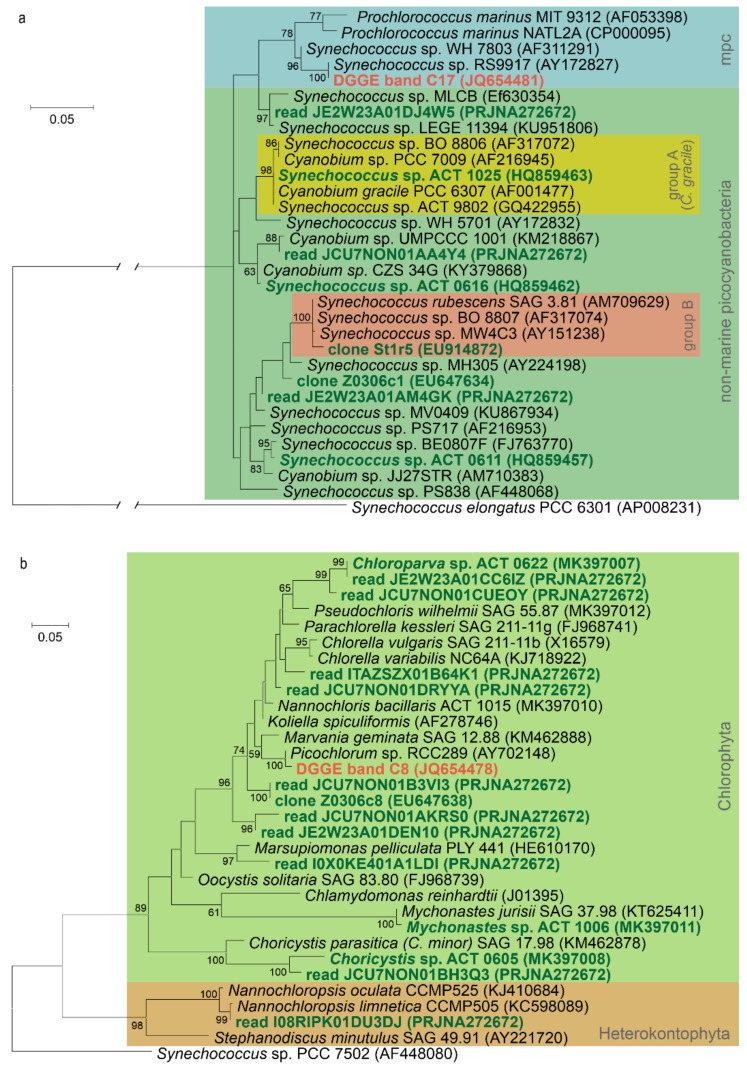
Maximum likelihood phylogenetic trees of picophytoplankton strains and the most abundant uncultured planktonic genotypes from the soda and hypersaline lakes of the Carpathian Basin based on 16S rRNA gene sequences. Sequences from the hypersaline lakes of the Carpathian Basin are marked with red, while sequences from soda lakes and pans are marked with green. Re-analysis of sequence data from the references listed in Table 3. The cyanobacterial phylogenetic tree (**a**) is based on 581, the plastid tree (**b**) is based on 382 nucleotide positions, and, in both cases, the Kimura 2-parameter substitution model was applied. For methodological details of phylogenetic tree construction, see Kalwasińska et al. [88]. GenBank codes and BioProject IDs are given in parentheses. Bootstrap values higher than 50 are shown at the nodes. Abbreviation: mpc—marine picophytoplankton clade.

Taxonomic studies on the picophytoplankton communities of the soda and saline lakes of the Carpathian Basin revealed a diverse composition, both in the case of picocyanobacteria and picoeukaryotic algae (Table 3, Figure 4). In general, more taxa were detected in the soda lakes than in the hypersaline lakes, confirming that increasing salinity reduces taxonomic diversity. In the hypersaline lakes, practically one cyanobacterium (marine *Synechococcus*, clade VIII) and one eukaryotic alga (*Picochlorum*) represent the APP community [4,17,87,88,89]. In the soda lakes, more diverse picocyanobacterial genotypes were detected, all belonging to various non-marine groups of *Synechococcus* (group A = *Cyanobium gracile*, group B and others) [1,11,14,87]. Eukaryotic APP consisted mainly of trebouxiophycean taxa (such as *Chloroparva*, *Choricystis* and *Mychonastes*), and genotypes without closely related cultured representatives [15,16,87,88,89,90,91,92,93]. Additionally, some rarely detected genera (*Marsupiomonas* and *Nannochloropsis*) having pico-sized cells also contributed occasionally to the picophytoplankton communities in these soda lakes [8,87]. The taxonomic composition of APP communities changes seasonally [87,92,93], while the environmental factors determining these shifts are currently unknown.

Comparing the main taxa detected in the soda and hypersaline aquatic environments of the Carpathian Basin, closely related uncultured genotypes and isolates were revealed also from similar habitats (e.g., Soap Lake, Mono Lake and brackish waters; for further details, see Figures S2 and S3 in Szabó et al. [83]). The worldwide occurrence of soda and saline lakes coincides with this observation, while it should be noted that a new chlorophyte picophytoplankton genus, *Chloroparva*, was first described from this region [15], and a high uncultured (unknown) fraction of the total APP community still waits for detailed taxonomic and physiological characterization.

## 7. Conclusions

In the Carpathian Basin, a huge chemical diversity of lakes exists, with a remarkable variety of saline lakes, including soda lakes and NaCl-dominated salt lakes. The extreme conditions (high turbidity and salinity) present in these aquatic habitats result in the predominance of picophytoplankton. Other physicochemical characteristics (e.g., the amount of CDOM and temperature) further modulate the composition and abundance of APP communities. The unique ecological role (i.e., high contribution to primary production) of these tiny planktonic photoautotrophs differs in the soda and hypersaline lakes of the Carpathian Basin from that in other shallow lakes, which raises questions for future studies, such as how top–down control (viral lysis, grazing pressure or the lack of it) influences the abundance and taxonomic composition of APP communities in these special ecosystems.

## Figures and Tables

**Table 1:** Selected environmental variables of the studied lakes. Abbreviations: TSS—total suspended solids, CDOM—coloured dissolved organic matter, TP—total phosphorous, APP—autotrophic picoplankton. In the case of hypersaline lakes, data from anoxic monimolimnion are not included. Data from the publications in Table S2 expanded with unpublished results.

Lake	TSS		CDOM		TP		Chlorophyll *a*		APP Abundance		APP Contribution	
	(mg/L)		(mg/L)		(mg/L)		(µg/l)		(10^6^ Cells/mL)		(%)	
	Range	Mean	Range	Mean	Range	Mean	Range	Mean	Range	Mean	Range	Mean
Hypersaline 1	1–16	4.4	-		0.5–0.7	0.5	6–104	28	0.02–7.3	1.5	8.5–95	49
Hypersaline 2	3–9	4.5			0.25–0.29	0.27	15–130	40	0.1–7.6	3.2	2–89	70
Hypersaline 3	8–57	33.5			0.3–0.5	0.4	44–430	240	0.4–7	2.9	65–99	88
Hypersaline 4	1.3–5	2.5			0.01–0.1	0.1	6–27	13	0–0.1	0.04	3–81	38
Hypersaline 5	0.8–1.5	1.1			3–9.5	5.5	0.08–0.09	0.09	0.1–0.5	0.12	3–89	51
Hypersaline 6	1–2.2	1.3			0.01–0.1	0.1	1–10	5.8	0–1.49	0.4	2–57	26
Hypersaline 7	1–19	5			0.01–0.1	0.1	1–89	27	0–0.98	0.29	2–96	59
Hypersaline 8	1–9	4.5			0.17–0.21	0.19	0–116	25.9	0–0.01	0.001	0–1.5	0.25
Hypersaline 9	1–1.3	1.2			0.3–12.5	4.5	0.4–550	110	0–27.8	2.4	0–100	64
Turbid soda 1	1–481	58	14–45	25	0.02–0.1	0.04	0.7–40	12	0.1–5.1	0.7	3–75	25
Humic soda 1	0.6–110	12	46–260	110	0.01–0.04	0.03	0.7–60	7.5	0–2.5	0.2	0–68	11
Humic soda 2	3–1090	125	390–13,000	4600	0.3–4	1.6	0–3150	260	0–14	0.7	0–60	7.8
Turbid soda 2	6–4520	930	350–3300	1600	0.15–2	0.77	1–400	70	0–64	12	0–100	79
Turbid soda 3	470–9600	3050	310–1300	810	2.6–17	8.5	2–1100	230	0–114	16	2–100	91
Turbid soda 4	73–7500	1000	130–800	385	0.5–13	5	3–700	140	0–146	18	0–100	74
Turbid soda 5	100–8700	1900	280–1400	640	1.5–9	5.2	2–1860	210	0–144	18	0–100	90
Turbid soda 6	155–8230	1700	130–950	400	2–22	7.5	2–2750	335	0–160	26	0–100	95

**Table 2:** Parameters of the lines fitted to the log_10_[APP abundance (cells/mL)] vs. log_10_[chlorophyll *a* concentration (µg/L)] relationships. The data used for the relationships of soda and hypersaline waters are from the publications in Table S2 expanded with unpublished results.

Ecosystem Type	Data Source	Slope			Intercept		
		Estimate	Lower	Upper	Estimate	Lower	Upper
			Bound of 95% Confidence Interval			Bound of 95% Confidence Interval	
Freshwaters	Bell and Kalff (2001)	0.74	0.52	0.96	4.16		
Shallow lakes with TSS-Org ≤ 50 mg/L	Somogyi et al. (2017)	0.33	0.05	0.60	5.05	4.80	5.30
Hypersaline lakes	Present study	0.89	0.66	1.12	4.23	3.90	4.56
Humic soda lakes	Present study	1.02	0.76	1.27	3.92	3.57	4.27
Turbid soda lakes	Present study	1.17	1.09	1.25	4.37	4.21	4.52

**Table 3:** List of APP taxa detected in the soda and saline lakes of the Carpathian Basin.

Taxon	Name of the Lake or Pan	Habitat Type	Reference
CYANOBACTERIA *			
Synechococcus, non-marine group A (*Cyanobium gracile* cluster)	L. Neusiedl—open water (S, U)	Soda (turbid)	[1,11]
Synechococcus, non-marine group B	L. Neusiedl—open water (U)	Soda (turbid)	[11]
*Synechococcus*, other non-marine	Böddi-szék (S, U), Büdös-szék (U), Kelemen-szék (U), Rusanda (U), Sós-ér (U), Zab-szék (S, U)	Soda (turbid), soda (humic)	[1,11,14,87]
*Synechococcus*, marine clade VIII	L. Cabdic (U), L. Tarzan (U), L. Ursu (U)	Hypersaline	[4,17]
CHLOROPHYTA			
*Chloroparva pannonica*	Böddi-szék (S)	Soda (turbid)	[15]
*Chloroparva* sp.	Böddi-szék (S), Sós-ér (U), Zab-szék (S, U)	Soda (turbid), soda (humic)	[8,15,87,88]
*Choricystis* sp.	Böddi-szék (S), Lake Fertő—inner pond (S), Zab-szék (S, U)	Soda (turbid)	[9,16,87]
Other trebouxiophycean taxa	L. Fehér (U), Slano Kopovo (U), Sós-ér (U), Zab-szék (U)	Soda (turbid), soda (humic)	[1,8,87]
*Marsupiomonas* sp.	Zab-szék (U)	Soda (turbid)	[8,87]
*Mychonastes* sp.	Böddi-szék (S), Lake Fertő—inner pond (S)	Soda (turbid)	[9,16,87]
*Picochlorum* sp.	L. Băilor (S), L. Băilor Cojocna (S, U), L. Cabdic (S, U), L. Durgău Cojocna (S), L. Ocnita-Avram Iancu (U), L. Tarzan (S), L. Ursu (U)	Hypersaline	[17,89], unpublished results
HETEROKONTOPHYTA			
*Nannochloropsis* sp.	Zab-szék (U)	Soda (turbid)	[87]

* Cluster designations according to Crosbie et al. [86] and Fuller et al. [87]. S—strain, U—uncultured genotype (amplicon sequence read, environmental clone or DGGE band sequence).

## Data Availability

The data used for the review can be found in the cited publications.

## Supplementary Materials

The following supporting information can be downloaded at: https://www.mdpi.com/article/10.3390/microorganisms10040818/s1, Figure S1: Map of the studied soda and hypersaline lakes; Figure S2: Selected environmental parameters of the studied soda and hypersaline lakes. Abbreviations: TSS (total suspended solids), CDOM (coloured dissolved organic matter), TP (total phosphorous). The average values are represented as black line. Out-of-range values are shown as dots; Figure S3: Abundance of picocyanobacteria (a) and picoeukaryotes (b) in the studied soda and hypersaline lakes (Re-analysis of data from references detailed in Table S1, expanded with unpublished results); Figure S4: Relationship between APP abundance and chlorophyll *a* concentration in the studied hypersaline (a), humic (b) and turbid (c) soda lakes (Re-analysis of data from references detailed in Table S1, expanded with unpublished results); Figure S5: Relationship between APP contribution to total phytoplankton biomass and chlorophyll *a* concentration in the studied hypersaline (a), humic (b) and turbid (c) soda lakes (Re-analysis of data from references detailed in Table S1, expanded with unpublished results); Figure S6: Maximum photosynthetic rate (a) and light utilization parameter (b) of a picoeukaryotic (EuAPP) and a picocyanobacterial (CyAPP) strain at different temperatures (data from Somogyi et al., 2009); Figure S7: Spectral distribution of mean water column irradiance (I_mean_) in a turbid and in a humic soda lake and *in vivo* absorbance spectra of a picoeukaryotic (EuAPP) and a picocyanobacterial (CyAPP) strain (Unpublished results); Figure S8: Growth rate of picoeukaryotic algal strains (a: *Choricystis* sp., b: *Mychonastes* sp., c: *Chloroparva pannonica*, d: *Picochlorum oklahomense*) isolated from freshwater (F), soda (S) and hypersaline (H) environments along with salinity. Isolates were grown on a Johnson Medium (Johnson et al., 1968) containing 0–200 g/L NaCl under 130 µmol/m^2^/sec in a 14:10 hour light: dark cycle at 26 °C (Unpublished results); Table S1: List of published papers on APP in soda and hypersaline lakes of the Carpathian Basin. Abbreviations: TS—Turbid soda lakes, HS—Humic soda lakes, H—Hypersaline lakes, NGS—next-generation DNA sequencing; List of investigated lakes, sampling strategy and selected Limnological characteristics. Abbreviations: H—Hungary, A—Austria, R—Romania, S—Serbia, EC—Electric conductivity, O—occasional sampling, R—regular sampling.
