# Supplementary material for: Where the Little Ones Play the Main Role—Picophytoplankton Predominance in the Soda and Hypersaline Lakes of the Carpathian Basin
Source: Microorganisms. 2022 Apr 14;10(4):818. doi: 10.3390/microorganisms10040818 (PMC9030754; doi:10.3390/microorganisms10040818)
Supplement: Supplementary file 1 [file microorganisms-10-00818-s001.zip › microorganisms-1638445-supplementary.pdf]

microorganisms

Supplementary materials

Table S1. List of published papers on APP in soda and hypersaline lakes of the Carpathian Basin. Abbreviations: TS – Turbid soda lakes, HS – Humic soda lakes, H – Hypersaline lakes, NGS - next-generation DNA sequencing.

Publication	Data type	Lake Category	Time period	Use in present study*
Vörös & V-. Balogh, 2003	APP abundance, contribution	TS	2000	-
Vörös et al. 2005	APP abundance, contribution	TS	2001	Figure 1, Table 1
Vörös et al. 2008	APP abundance	TS	2002, 2004	Figure 1, Table 1
Somogyi et al. 2009a	APP abundance	TS	2006-2007	Figure 1, Table 1
Felföldi et al. 2009	APP abundance, DGGE and cloning of environmental samples	TS, HS	2003-2005	Figures 1, 4; Tables 1, 3
Somogyi et al. 2010	APP abundance, contribution, cloning of environmental samples	TS, HS	2004	Figures 1, 4; Tables 1, 3
Somogyi et al. 2011a	APP abundance, contribution	TS, HS	2008-2009	Figure 1, Table 1
Somogyi et al. 2011b	New species description	TS	2006	Figure 4, Table 3
Felföldi et al. 2011	DNA analysis (isolated strains)	TS	2006	Figure 4, Table 3
Somogyi et al. 2012	DNA analysis (isolated strains)	TS	2005-2006	Table 3
Keresztes et al. 2012	DGGE of environmental samples	H	2010	Figure 4, Table 3
Somogyi et al. 2013	DNA analysis (isolated strains)	TS	2005	Figure 4, Table 3
Somogyi et al. 2014	APP abundance, contribution	H	2010, 2011	Figure 1, Table 1
Pálffy et al. 2014	APP abundance, photosynthesis, Cloning of environmental samples	TS	2012	Figure 1, Tables 1, 3
Máthé et al. 2014	DGGE of environmental samples	H	2009	Table 3
Mentes et al. 2014	APP abundance, Cloning of environmental samples	H	2010	Table 3
Somogyi et al., 2016	APP abundance, photosynthesis, DNA analysis (isolated strains)	TS, HS	2012, 2014	Figure 1, Table 1
Korponai et al. 2015	APP biomass, contribution	TS, HS	2013-2014	Figure 1, Table 1
Felföldi et al. 2016	APP abundance, DGGE of environmental samples	H	2013	
Szabó et al. 2017	NGS of environmental samples	TS, HS	2012	
Somogyi et al. 2017	APP biomass, contribution	TS, HS	2008-2009, 2013	Figure 1, Table 1
Felföldi 2020	combination of microscopic cell counts with NGS of environmental samples	TS	2013-2014	-
Szabó et al. 2020	APP abundance, NGS of environmental samples	TS, HS	2013-2014	Figure 4, Table 3

* all data are used in the text.

Table S2. List of investigated lakes, sampling strategy and selected Limnological characteristics. Abbreviations: H – Hungary, A – Austria, R – Romania, S – Serbia, EC – Electric conductivity, O – occasional sampling, R – regular sampling.

Lake		Category	Coordinates	Sampling strategy		Surface	Mean depth	EC		pH	
								range	mean	range	mean
						(ha)	(cm)	(mS cm^{-1})			
L. Cabdic	R	Hyper-saline 1	N47°07.712' E23°51.900'	2010 – 2011	O (n= 5)	0.15	890	84-195	97	7.3-8..9	7.9

L. Băilor	R	Hyper-saline 2	N46º55.913' E23º54.073'	2010 – 2011	O (n= 7)	0.06	250	48-108	75	8.1-8.8	8.5
L. Băilor Co-jocna	R	Hyper-saline 3	N46°44.907' E23°50.441'	2010 – 2011	O (n= 3)	0.24	350	89-133	118	8.2-8.6	8.4
L Durgău Co-jocna	R	Hyper-saline 4	N46°44.836' E23°50.442'	2010 – 2011	O (n= 4)	0.24	920	69-142	133	7.4-8.6	8.1
L. Tarzan	R	Hyper-saline 5	N46º34.472' E23º48.549'	2010 – 2011	O (n= 10)	0.36	490	32-61	42	7.1-9.2	7.9
L. Ocnei	R	Hyper-saline 6	N46°35.158' E23°47.282'	2010 – 2011	O (n= 7)	0.21	1200	68-280	123	7.1-8.9	8.2
L. Rotund	R	Hyper-saline 7	N46°35.099' E23°47.210'	2010 – 2011	O (n= 4)	006	330	27-100	72	8.1-8.6	8.4
L. Fără Fund	R	Hyper-saline 8	N45º52.578' E24º04.064'	2010 – 2011	O (n= 7)	0.17	600	101-330	231	6.4-9.4	7.7
L. Ursu	R	Hyper-saline 9	N46º36.226' E25º05.164'	2013 – 2015	O (n= 17)	4		20-348	160	6.6-9.2	8.1
L. Neusied, open water	H/A	Turbid soda 1	N47°46.228' E16°43.298'	2004, 2008-2009,2015-2016	R (n= 49)	13800	130	1.7-3	2.2	8.3-9.7	9.0
L. Neusiedl, reed belt	H/A	Humic soda 1	N47°41.100' E16°42.173'	2004, 2008-2009,2015-2016	R (n= 65)	17200	110	1-5	2.3	7.8-9.3	8.6
Sósér pan	H	Humic soda 2	N46°47.341' E19°8.679'	2013-2014, 2016-2017	R (n= 35)		20	1.3-23.6	5.3	8.0-10.1	9.1
Fehér-szék pan	H	Turbid soda 2	N46°48.448' E19°11.221'	2001, 2013-2014	R (n= 33)	10	25	0.9-12.7	5.2	8.3-10	9.1
Büdös-szék pan	H	Turbid soda 3	N46°51.980' E19°10.153'	2005-2007, 2014	R (n= 26)	43	10	1.6-9.2	5.1	8.7-9.9	9.4
Böddi-szék pan	H	Turbid soda 4	N46°46.061' E19°8.726'	2005-2007, 2013-2014	R (n= 50)	198	12	1.4-31	8.7	8-10	9.4
Kelemen-szék pan	H	Turbid soda 5	N46°47.542' E19°10.647'	2001-2002, 2006-2007, 2013-2014	R (n= 43)	190	23	1.5-22.8	7.3	8.7-10.2	9.4
Zab-szék pan	H	Turbid soda 6	N46°50.190' E19°10.283'	2001-2002, 2006-2007, 2013-2017	R (n= 57)	182	20	2.1-36	8.6	8.9-10.2	9.6
Slano Kopovo pan	S	Turbid soda 7	N45°37,548' E20°12,521'	2005	O (n=1)	119	30		8.9		3.22
Rusanda pan	S	Turbid soda 8	N45°30,742' E20°18,113'	2005	O (n=1)	161	20		9.3		10.4

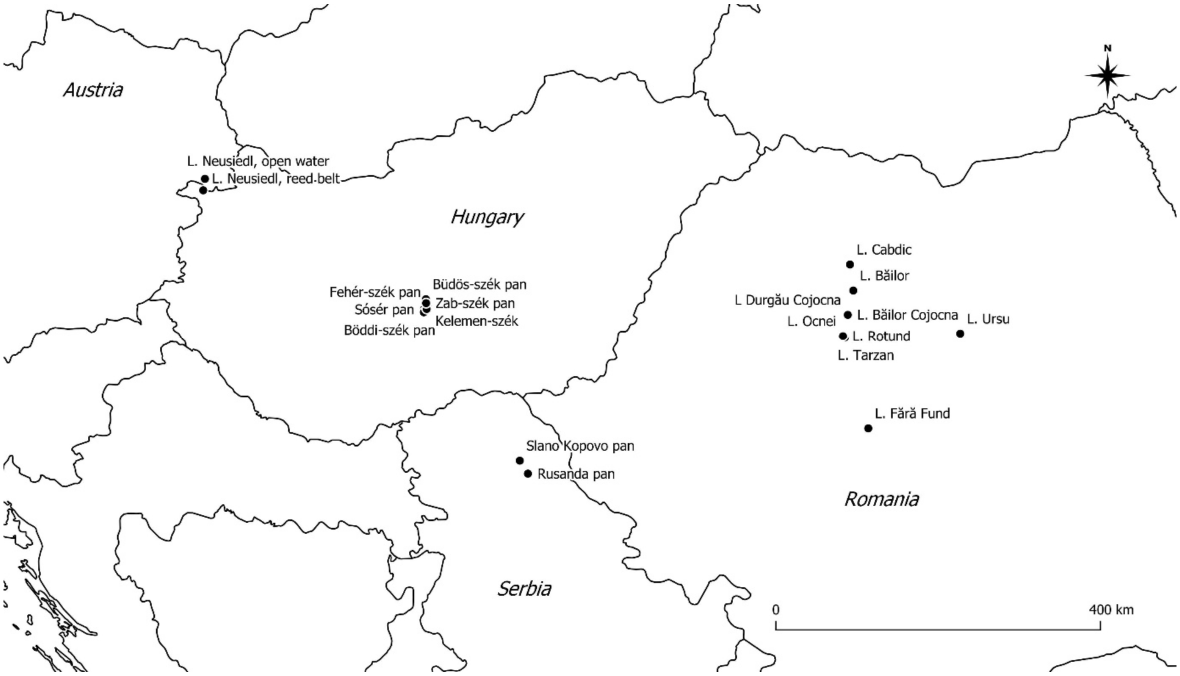

Figure S1. Map of the studied soda and hypersaline lakes.

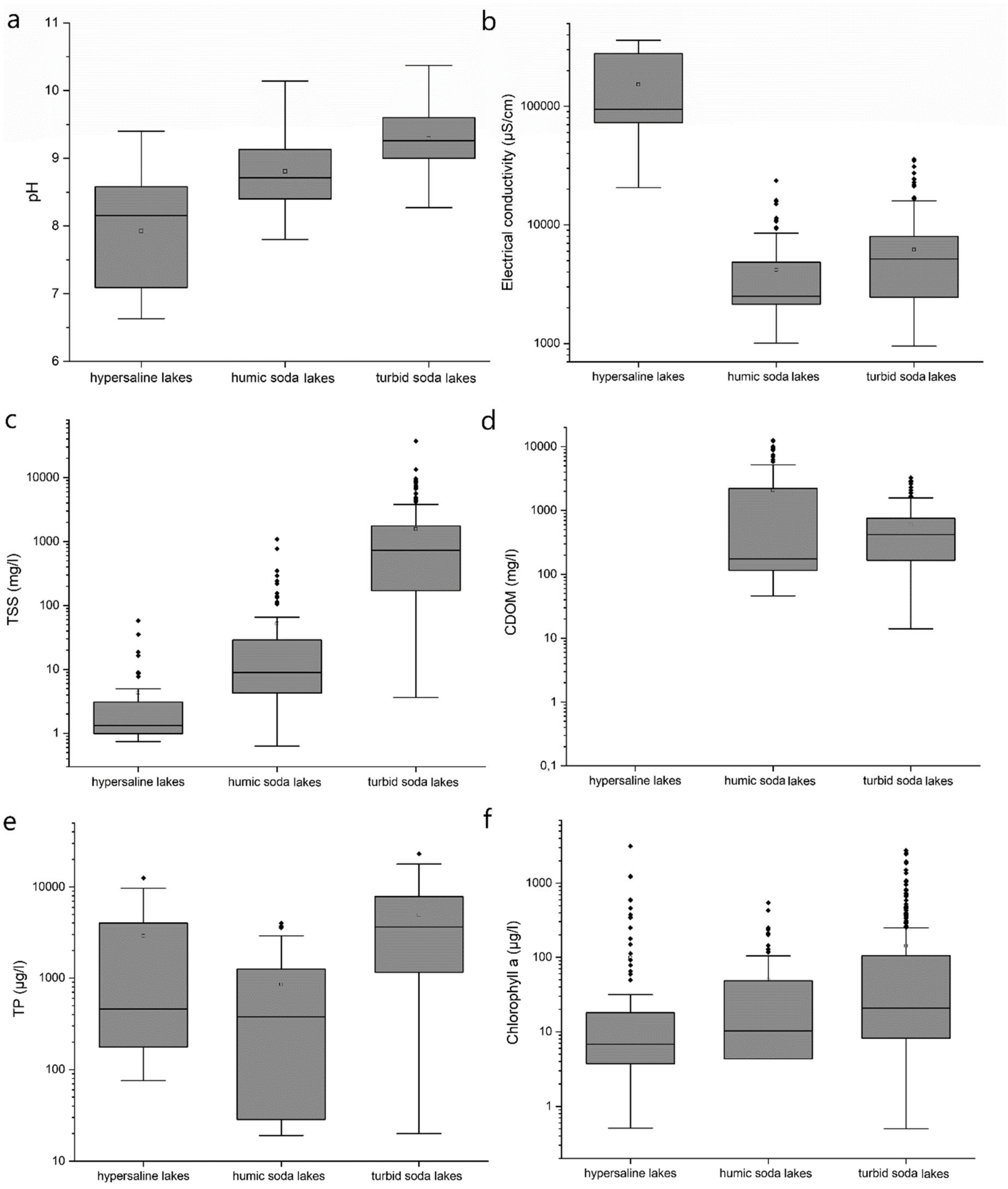

Figure S2. Selected environmental parameters [pH (a), electrical conductivity (b), TSS (c), CDOM (d), TP (e) and chlorophyll a (f) concentration] of the studied soda and hypersaline lakes. Abbreviations: TSS (total suspended solids), CDOM (coloured dissolved organic matter), TP (total phosphorous). The average values are represented as black line. Out-of-range values are shown as dots.

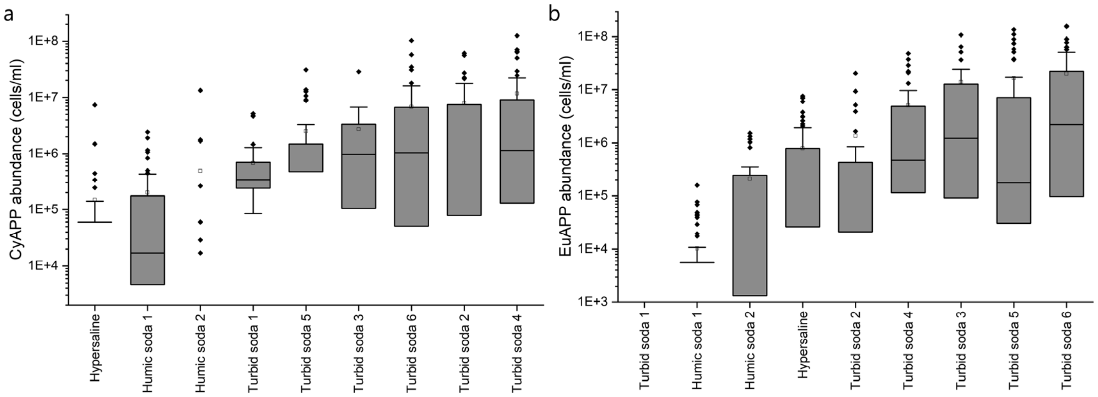

Figure S3. Abundance of picocyanobacteria (a) and picoeukaryotes (b) in the studied soda and hypersaline lakes (Re-analysis of data from references detailed in Table S1, expanded with unpublished results).

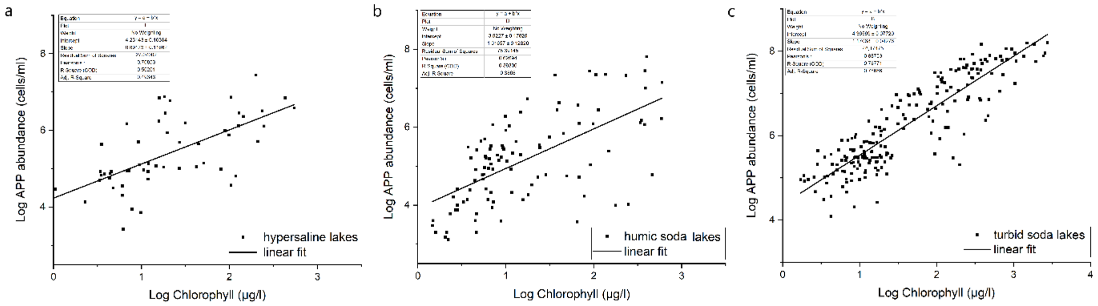

Figure S4. Relationship between APP abundance and chlorophyll *a* concentration in the studied hypersaline (a), humic (b) and turbid (c) soda lakes (Re-analysis of data from references detailed in Table S1, expanded with unpublished results).

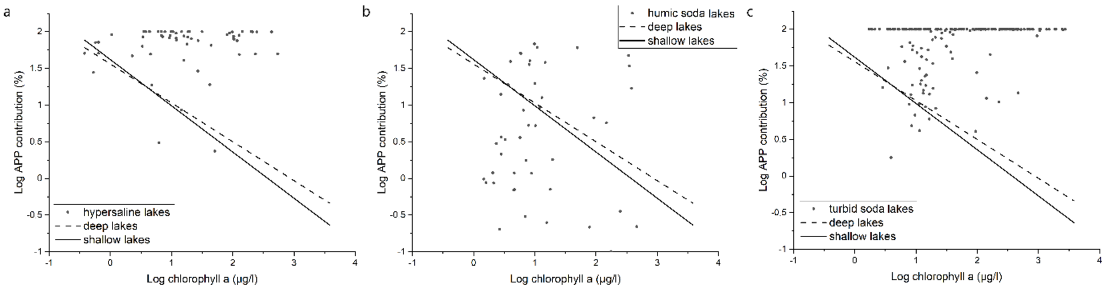

Figure S5. Relationship between APP contribution to total phytoplankton biomass and chlorophyll *a* concentration in the studied hypersaline (a), humic (b) and turbid (c) soda lakes (Re-analysis of data from references detailed in Table S1, expanded with unpublished results).

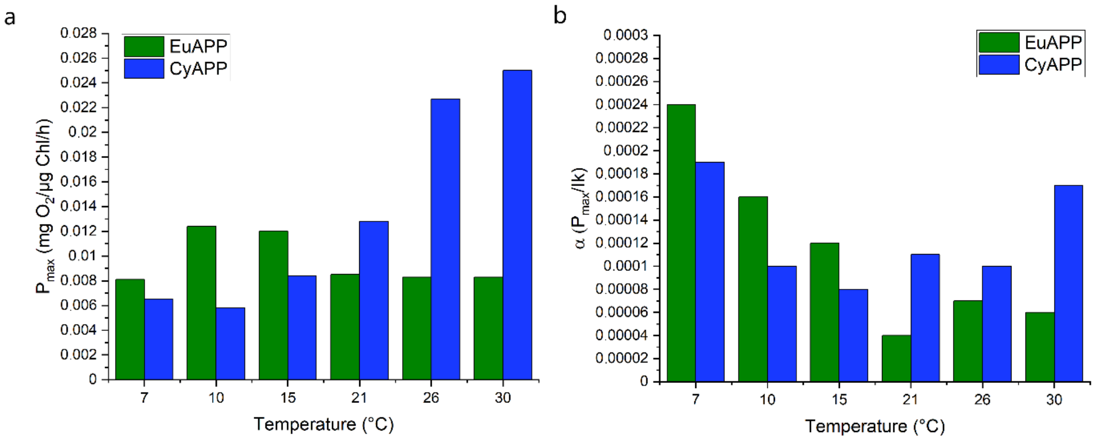

Figure S6. Maximum photosynthetic rate (a) and light utilization parameter (b) of a picoeukaryotic (EuAPP) and a picocyanobacterial (CyAPP) strain at different temperatures (data from Somogyi et al., 2009).

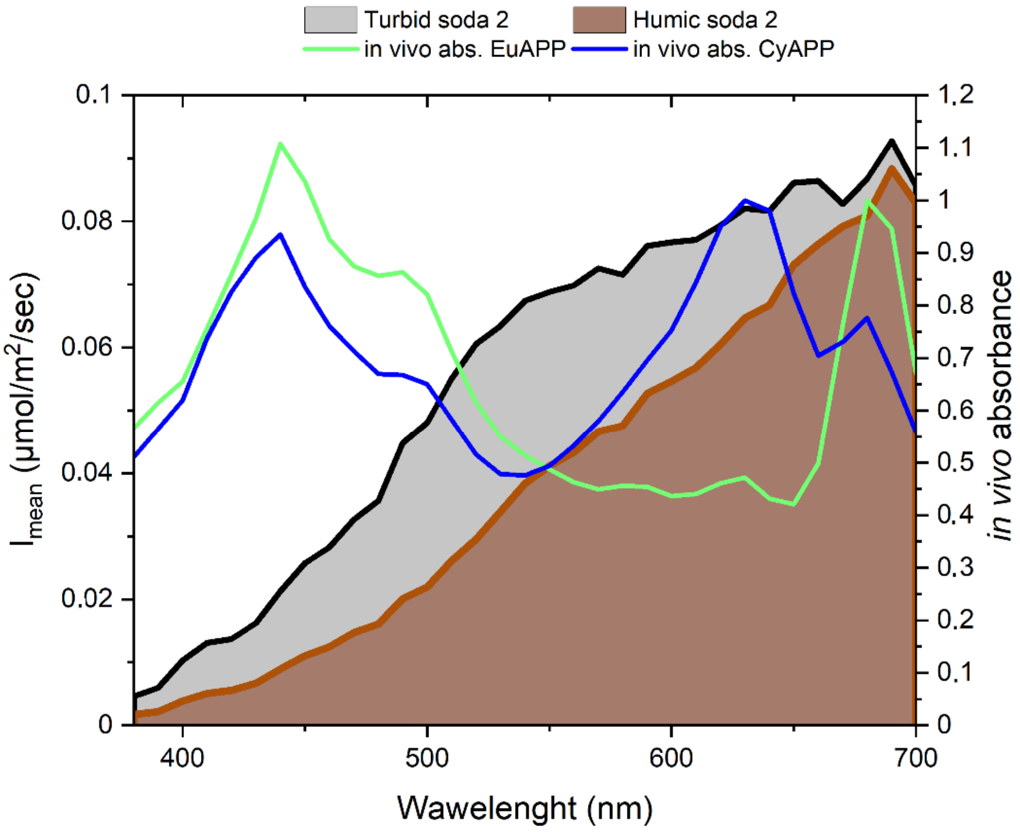

Figure S7. Spectral distribution of mean water column irradiance (I_{mean}) in a turbid and in a humic soda lake and *in vivo* absorbance spectra of a picoeukaryotic (EuAPP) and a picocyanobacterial (CyAPP) strain (Unpublished results).

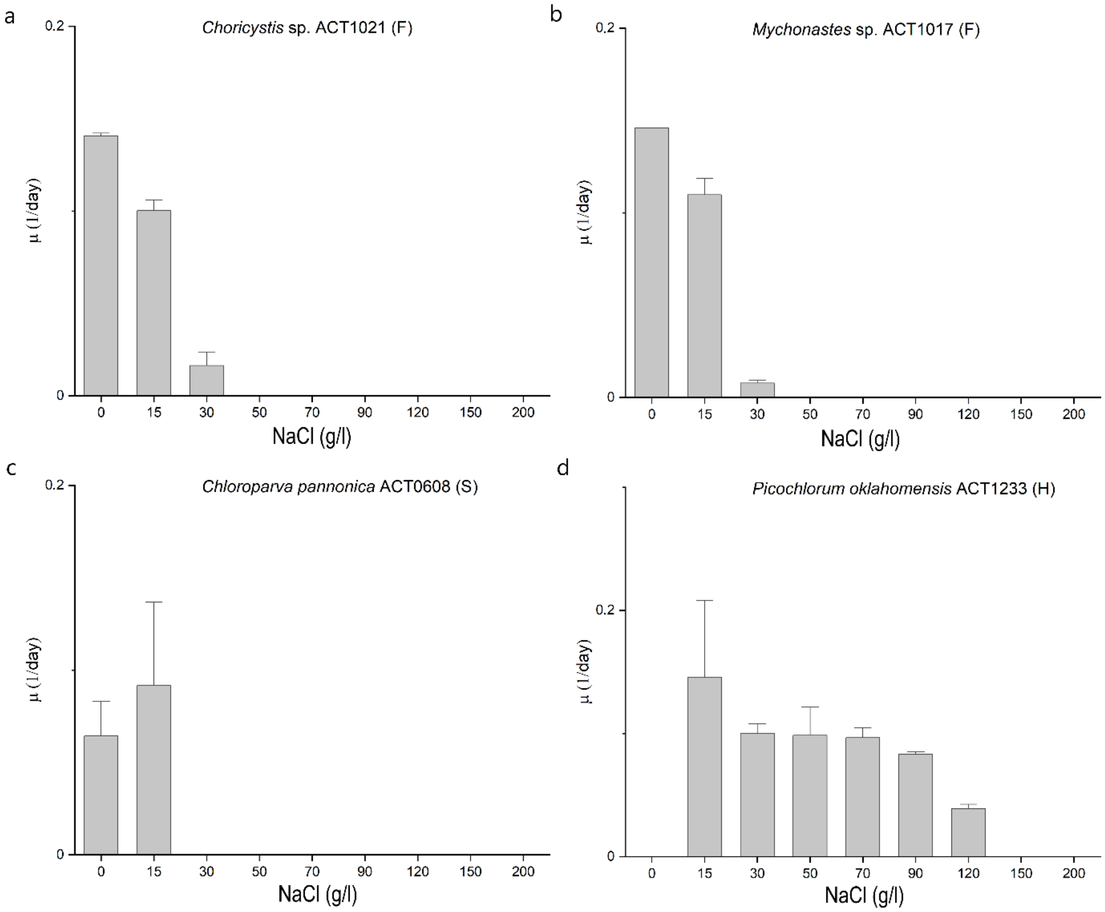

Figure S8. Growth rate of picoeukaryotic algal strains (a: *Choricystis* sp., b: *Mychonastes* sp., c: *Chloroparva pannonica*, d: *Picochlorum oklahomense*) isolated from freshwater (F), soda (S) and hypersaline (H) environments along with salinity. Isolates were grown on a Johnson Medium (Johnson et al., 1968) containing 0 - 200 g/l NaCl under 130 µmol/m^2/sec in a 14:10 hour light: dark cycle at 26 °C (Unpublished results).
